# Effects of a Whole-School Prevention Program Targeting Mental Health and Nonsuicidal Self-Injury in Swedish Adolescents: A Cluster-Randomized Experimental Study with Longitudinal Follow-Up

**DOI:** 10.1007/s10964-025-02251-3

**Published:** 2025-09-11

**Authors:** Erik Aspeqvist, Laura Korhonen, Örjan Dahlström, Hedvig Andersson, Imke Baetens, Paul Plener, Maria Zetterqvist

**Affiliations:** 1 Center for Social and Affective Neuroscience, Department of Biomedical and Clinical Sciences, Linköping University, Linköping, Sweden; 2 Barnafrid, Swedish National Center on Violence Against Children, Department of Biomedical and Clinical Sciences, Linköping University, Linköping, Sweden; 3 Department of Child and Adolescent Psychiatry in Linköping, Region Östergötland, Linköping, Sweden; 4 Department of Behavioural Sciences and Learning, Linköping University, Linköping, Sweden; 5 Athletics Research Center, Linköping University, Linköping, Sweden; 6 Brussels University Consultation Center (BRUCC), Department of Psychology, Vrije Universiteit Brussel, Brussels, Belgium; 7 Department of Child and Adolescent Psychiatry, Medical University of Vienna, Vienna, Austria; 8 Comprehensive Center for Pediatrics (CCP), Medical University of Vienna, Vienna, Austria; 9 Department of Child and Adolescent Psychiatry and Psychotherapy, University of Ulm, Ulm, Germany

**Keywords:** Nonsuicidal self-injury, Self-harm, Prevention, School, Adolescence, Mental health

## Abstract

Rising rates of mental health problems and nonsuicidal self-injury (NSSI) among adolescents highlight the need for preventive interventions and the lack of evidence regarding such measures. To date, few studies have investigated school-based prevention programs targeting NSSI. In this study, a whole-school preventive intervention was carried out at Swedish lower secondary schools and evaluated in a cluster-randomized controlled trial. The whole-school intervention included classroom-based modules focusing on mental health and NSSI directed at students, psychoeducational webinars on NSSI directed at parents and teachers, and a two-day workshop on NSSI and suicidality for school health staff. Data were collected from students (*N* = 183, age *M* = 14.17, *SD* = 0.55, 58% female) at baseline and three- and six-month post-intervention follow-ups. Analyses revealed a significant decrease in three-month NSSI frequency and a significant difference in mental health-related stigma awareness in the intervention group compared to controls. Regarding other outcomes (NSSI onset, attitudes toward help-seeking, perceived social support, health-related quality of life, emotion regulation and self-criticism), no significant effects were found. Effects moderated by gender and history of NSSI were found, underscoring that the outcomes of universal prevention are not always uniformly distributed. Main conclusions were that whole-school prevention can be effective in reducing NSSI frequency as well as affecting the awareness of mental health-related stigma.

## Introduction

Over the past decade, several reports have pointed to the impact of mental health problems on youth worldwide (Castelpietra et al., 2022; Erskine et al., 2015), as well as the high prevalence of nonsuicidal self-injury (NSSI) among adolescents (Aspeqvist, Andersson, et al., 2024). This underscores the importance of mental health prevention, where schools are key arenas for reaching children and adolescents (Hoover & Bostic, 2021). However, interventions deployed in schools are rarely supported by scientific evidence (Laurens et al., 2021) and more research is needed on the effects of mental health prevention (World Health Organization, 2020), This study aimed to quantitatively investigate the effects of a mental health preventive intervention program targeting adolescents in Swedish lower-secondary schools. The study employed a cluster-randomized, controlled experimental design to investigate the effects of the intervention.

### Adolescent Mental Health

In 2019, almost 19.8% of European children and youth (ages 10 to 24) experienced mental or substance use disorders, with an increasing trend since 1990. Anxiety and depression were associated with the highest numbers of Years Lived with Disability (YLDs; Castelpietra et al., 2022). Most mental disorders have their onset before the age of 20 (McGrath et al., 2023), and mental conditions were the leading cause of disability among European children and youth (Castelpietra et al., 2022). This situation has led some to describe the current situation as a youth mental health crisis and to propose reforms in several areas of society to address mental health issues (McGorry et al., 2024).

### Nonsuicidal Self-injury

An important aspect of mental health is nonsuicidal self-injury. NSSI is defined as the intentional, self-directed damage to body tissue, which is performed without suicidal intent and not socially sanctioned (International Society for the Study of Self-Injury, 2025). Previously believed to be rare and primarily affecting troubled young girls (Chaney, 2017), NSSI is, in fact, a widespread phenomenon. It affects around 18% of adolescents (21% of girls and 14% of boys) in community samples globally, and is twice as common in adolescent females than in males in North America and Europe (Moloney et al., 2024). In Sweden, up to 40% of adolescents in community samples report that they have attempted the behavior at least once (Aspeqvist, Andersson, et al., 2024). The onset of NSSI typically occurs between the ages of 12 to 14, and the behavior is most common around the ages of 15 to 17 (Plener et al., 2015). NSSI is closely associated with emotion regulation difficulties (Wolff et al., 2019) and self-criticism (Zelkowitz & Cole, 2019), and emotion regulation and self-punishment are commonly reported functions underlying the behavior (Taylor et al., 2018). The growing body of literature investigating different trajectories of NSSI suggests the potential for considering different subtypes of NSSI engagement patterns, as well as associated functions and predictors of the behavior (Barrocas et al., 2015; Nakar et al., 2016; Tilton-Weaver et al., 2023).

Engagement in NSSI is a risk factor for various adverse outcomes (Daukantaite et al., 2020b). These outcomes include poor psychosocial well-being (Muehlenkamp et al., 2013), psychiatric disorders (Wilkinson et al., 2018) and suicide attempts (Ribeiro et al., 2016). For these reasons, it has been suggested that greater efforts to prevent NSSI are needed (Lloyd-Richardson et al., 2023).

### Mental Health Prevention

As described above, mental health promotion and prevention efforts are increasingly employed across society. Prevention efforts are typically categorized in three groups: universal, selective, and indicated (Mrazek & Haggerty, 1994). There is also similar, but not entirely translatable, terminology specifying primary, secondary and tertiary levels of prevention (Caplan, 1964; Gordon, 1983). Universal prevention is intended to reach entire populations and has the advantage of broad benefits, although this may come at a higher cost in terms of time and resources (Le et al., 2021). Selective prevention focuses on specific at-risk groups, potentially achieving better cost-effectiveness but relies on accurately identifying those groups (Cuijpers et al., 2021). Indicated prevention is designed to reach those who are already affected in some manner or at high risk and seeks to mitigate further negative consequences. Besides the issues mentioned above, key questions in mental health prevention are the effectiveness of the interventions over shorter and longer time spans, implementation, and efficacy (Fusar-Poli et al., 2021). Regarding children and adolescents, the timing of preventive interventions is another critical concern as there are sensitive developmental stages where both risk and protective factors may have a greater impact (Arango et al., 2018).

Knowledge of the effects in population subgroups is important in prevention research (Laurenzi et al., 2023). However, a recent meta-analysis of school prevention notes that knowledge is currently lacking on the effects among subgroups of students (Cipriano et al., 2023). Recommendations have therefore been put forward to include demographic variables such as gender, for example, for moderation analyses of effects. For example, barriers to help-seeking and stigma have been assumed to be more pronounced in males (Galdas et al., 2005).

### School-Based Preventive Intervention

Schools have been identified as a key area for mental health prevention since they are central to the lives of almost all children and typically have some health organization in place (Hoover & Bostic, 2021). Raising awareness and promoting mental health in schools are essential components of future youth mental healthcare (McGorry et al., 2024). Students generally hold positive attitudes toward including mental health in school activities (Aspeqvist, Münger, et al., 2024; Cortina et al., 2021). In recent decades, research on school-based preventive interventions has gained interest (see reviews in Singh et al., 2022; Weare & Nind, 2011). School-based mental health prevention targets a range of outcomes, including mental health literacy, help-seeking, mental health stigma, and peer support (Singh et al., 2022). Health-related quality of life (HRQoL), a multidimensional construct including a range of factors related to how a child’s health impacts their life and well-being, is often used to assess outcomes in a broader sense than specific health domains (Befus et al., 2023).

Prevention focusing on interpersonal skills, emotion regulation, and alcohol and drug education has been found to be effective (Skeen et al., 2019). A class of interventions labeled Social and Emotional Learning (SEL) combines a focus on intrapersonal and psychological matters, such as emotion regulation, with interpersonal social skills. Large meta-analyses (Cipriano et al., 2023; Durlak et al., 2011) as well as long-term follow-up studies (Taylor et al., 2017) have established that SEL interventions, although heterogeneous, have beneficial effects across a range of outcomes. Further, although adolescent suicidality is not a typical focus in SEL type interventions (Cipriano et al., 2023), this has been targeted in the Youth Aware of Mental Health (YAM) program, which was found to have an effect on the rate of suicide attempts in a large-scale randomized controlled trial across several European countries (Wasserman et al., 2012; Wasserman et al.). YAM integrates psychoeducation with role-playing sessions over five classroom sessions led by two trained instructors (Wasserman et al., 2012).

While universal school-based mental health prevention programs are generally beneficial (Werner-Seidler et al., 2021), the effect sizes are small, and further research on what predicts responses to universal, selective, and indicated prevention is recommended (Singh et al., 2022). Thus, more research is needed to understand the effects beyond population means and to move toward a more fine-grained understanding of what works for whom.

Regarding NSSI, there are recommendations for how schools should respond (Hasking et al., 2016; Heath & Toste, 2010), and a handful of studies have reported on the effects of school-based preventive interventions. It has been argued that NSSI prevention should target early adolescence, since the behavior reaches its peak prevalence around age 15 to 17 (Buerger et al., 2022; Plener et al., 2015). The Signs of Self-Injury program, developed by (Jacobs et al., 2009) was the first to target NSSI specifically. It was found to be feasible, with no iatrogenic effects, and to have a positive influence on NSSI knowledge and attitudes among high school students (age M = 16.07, SD = 1.32; Muehlenkamp et al., 2010). Baetens et al. (2020) conceptualized and studied an NSSI-specific educational module (KRAS) that was added to a mental health program for adolescents (*N* = 651, ages 11–15). It was found to be feasible, without iatrogenic effects. Additionally, school-based peer education focusing on NSSI had positive effects on emotion regulation, self-esteem, and other outcomes (Cipriano et al., 2021). A social-cognitive educational program in an all-female adolescent sample (*N* = 191, ages 15–17) was reported to have effects on the participants’ intention to stop or reduce their engagement in NSSI (Zare et al., 2023). Finally, a recent study reported a significant reduction in NSSI frequency, as well as effects on several secondary outcomes in a sample of secondary school students (*N* = 124, ages 11–14; Baetens et al., 2024) following participation in a four-hour universal prevention program including specific content targeting NSSI. Besides prevention programs for participating youth, educational efforts have targeted school staff to increase their knowledge and facilitate positive management of NSSI (Groschwitz et al., 2017; Lloyd-Richardson et al., 2020). Finally, both mental health issues and NSSI are associated with stigma (Ahad et al., 2023; Burke et al., 2019). Stigma is a barrier to help-seeking (Barrow & Thomas, 2022) and disclosing of NSSI (Rosenrot & Lewis, 2018), which are both factors related to NSSI cessation (Whitlock et al., 2015). Hence, stigma is important to target when addressing mental health and NSSI in adolescents.

In sum, the last decade has seen an increase in school-based NSSI prevention research. As evident from the literature review above, only a handful of studies have investigated preventive interventions focusing on NSSI, and none have a whole-school prevention concept. There is a shortage of universal prevention efforts targeting NSSI (Bürger et al., 2023), and more research is needed to investigate the effects of such efforts (Petrovic et al., 2023).

## Current Study

Mental health problems and NSSI in adolescents are a pressing concern, but strong evidence for addressing these issues preventively in schools is lacking. The primary aim of the present study was to investigate whether a whole-school preventive intervention program targeting mental health, NSSI, stigma, help-seeking and support can delay the onset of NSSI and reduce its frequency among adolescents, at an age where NSSI has yet to reach its peak frequency. Additionally, the study aimed to investigate the effects on social support, stigma, health-related quality of life factors, emotion regulation difficulties, self-criticism, and attitudes toward help-seeking as these are all important factors related to NSSI and mental health prevention. Based on the previous research described above, the study hypotheses were that the interventions would have effects delaying the onset of NSSI and reducing NSSI frequency. Further, it was expected that the intervention would lead to improvements in social support, self-criticism, stigma, help-seeking, health-related quality of life factors and difficulties in emotion regulation for the intervention group compared to controls. A further aim was to explore the effects specific to subgroups (gender and NSSI history). These subgroup analyses were exploratory without specific hypotheses.

## Methods

### Procedures

#### Recruitment

The present study followed a cluster randomized waitlist control paradigm. During the experimental phase, some schools were assigned to receive the whole-school intervention, while others served as controls. The control schools received the same intervention package later. The study was conducted in schools located in several municipalities in Östergötland County, Sweden. Recruitment of participating schools was initiated during the fall of 2021, when the research group contacted the principals of lower secondary schools (using a convenience sampling approach) and invited them to participate. Subsequently, caregivers of 7th and 8th-grade students in schools whose principals accepted participation received written information about the study along with consent forms. For a student to participate in the study, written informed consent from both the caregivers and the students themselves was required.

#### Data Collection

Three waves of measurements were conducted between January and March 2022 (baseline measurement), May and June 2022 (3-month post-intervention measurement), and August 2022 (6-month follow-up measurement). Data were collected using the REDCap digital survey platform (Harris et al., 2009). Participants completed a survey battery using school laptops at designated times at their schools, with study staff present. School health staff were notified beforehand to be available in case their services were needed by students during study participation, and participants received written information on where to seek professional help.

The study was approved by the Swedish Ethical Review Authority (2021-01699, 2021-05049). The study was funded by a grant from the Swedish Research Council (2018-05820), preregistered on ClinicalTrials.gov (ID NCT05935345).

### Participants

#### Schools

**Fig. 1:**
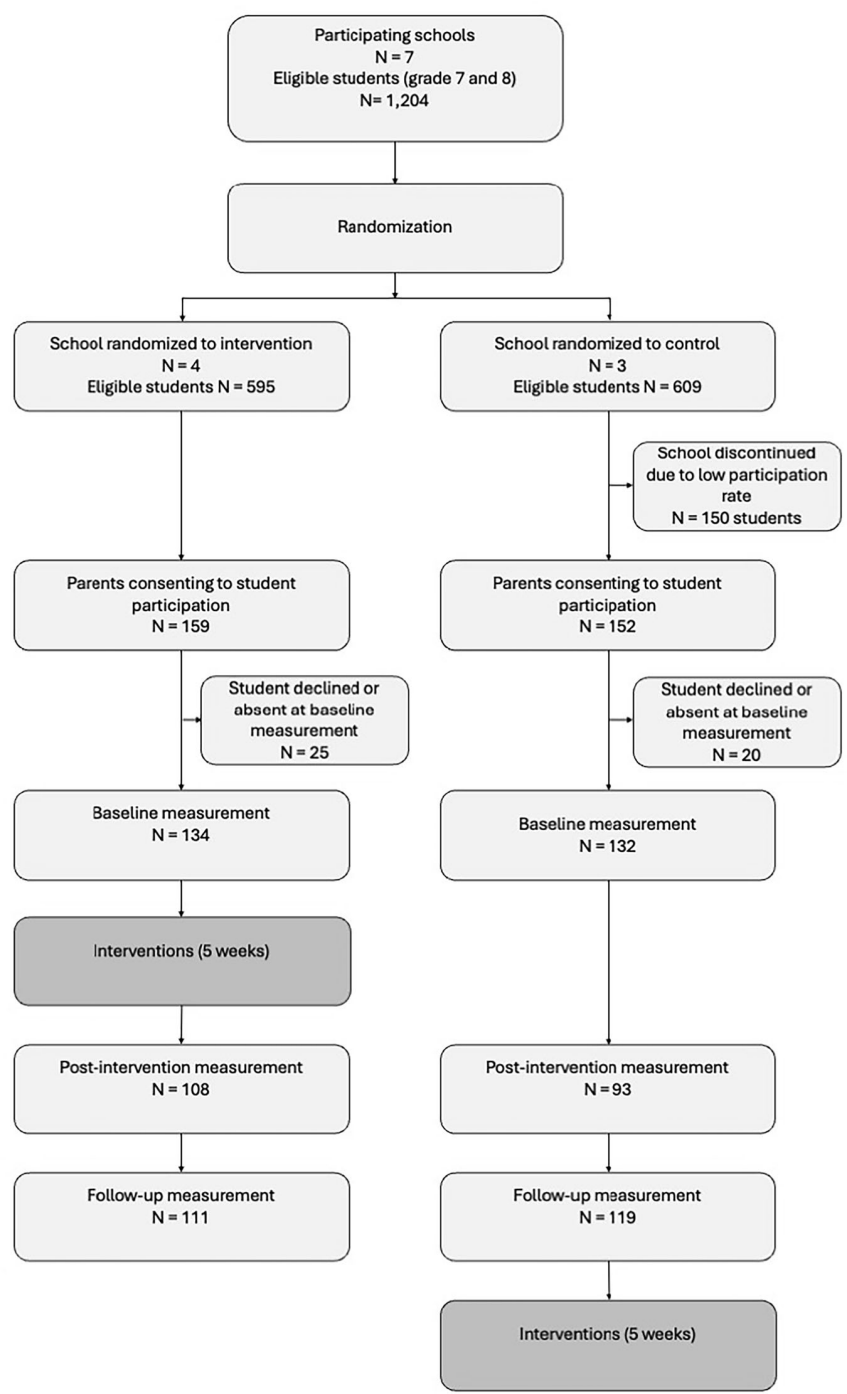
Flowchart of Recruitment, Randomization, Measurements and Interventions

Initially, seven schools were recruited for the project, and project staff gathered participants from these schools (see Fig. 1). Due to a low participation rate (approximately 5% of parents giving informed consent) at one of the schools, it was decided not to collect data there, and all eligible participants (*n* = 150) were excluded. Thus, six schools participated in the project, five municipally administered and one privately managed. Two schools were located in a medium-sized city municipality, two in smaller city municipalities, and two in rural municipalities. Five schools were of medium size (enrolling 120–238 students in grades 7 and 8), while one was smaller (fewer than 100 students in grades 7 and 8). According to available demographics (SALSA; Skolverket [The Swedish National Agency for Education], n.d.), the medium-sized schools had a somewhat lower share of students with foreign backgrounds (ranging from 6 to 22%, while the Swedish mean is 27%) and a proportion of students whose parents had received post-secondary education at or slightly below the average level (45–63%, with the mean for all Swedish schools being 62%). The smaller school had a higher proportion of students with foreign backgrounds (over 50%). All schools had a similar proportion of female students (41–49%).

#### Adolescents

To be eligible for participation, students needed to be enrolled in a regular school class, specifically in grades seven or eight, following the standard Swedish curriculum. In the six participating schools, 1054 students were eligible to participate in the study. Of these, 267 agreed to take part after receiving written informed consent from both caregivers, and 266 proceeded to baseline data collection. The calculated participation rate was 25.3%. Participant demographics are presented in Table 1.

**Table 1:** Participants Demographics

Characteristics	Full sample *N* = 266 *n* (%)	Study sample *n* = 183 *n* (%)	Intervention group *n* = 97 *n* (%)	Control group *n* = 86 *n* (%)
Age, years (*M*, *SD*)	14.21 (0.56)	14.17 (0.55)	14.16 (0.54)	14.17 (0.57)
Gender				
Girls	155 (58.27)	106 (57.92)	55 (56.7)	51 (59.3)
Boys	105 (39.47)	72 (39.34)	40 (41.24)	32 (37.21)
Non-binary	6 (2.26)	5 (2.73)	2 (2.06)	3 (3.49)
Living with both parents	191 (71.8)	133 (72.68)	69 (71.13)	64 (74.42)
Region of origin				
Sweden	253 (95.11)	176 (96.17)	92 (94.85)	84 (97.67)
Other European country	7 (2.63)	5 (2.73)	4 (4.12)	1 (1.16)
Outside of Europe	6 (2.26)	2 (1.09)	1 (1.03)	1 (1.16)

Of the 266 participants included at baseline, 201 (75.6%) participated in the post-intervention data collection and 230 (86.5%) at follow-up. Thus, attrition rates were 24.4% at post-measurement and 13.5% at follow-up. The number of participants who had data from all three time points and was included in the analyses in the present study was 183 (68.8% of the full sample).

### Whole-school Intervention

In the experimental phase, the intervention schools received a whole-school intervention - a package of interventions intended to reach students, school staff and parents. This included universal interventions available to all members of the respective populations, along with targeted interventions provided to a specific subset. The current study mainly focuses on the effects of the two universal interventions delivered directly to adolescents, i.e., YAM and KRAS. The schools offered YAM and KRAS to all students during school hours. Student attendance was not registered during classroom sessions. The first was the YAM program (Wasserman et al., 2012), designed to address mental health and suicidality in adolescents aged 14 to 16, featuring psychoeducation and interactive role-play sessions. YAM is manual-based and carried out by instructors, trained in the method and manual, over the course of five hour-long classroom sessions, typically delivered over a time-period of three weeks. When a class is participating in YAM, educational posters are placed in the classrooms and students receive a booklet to take home. Sessions include interactive lectures on mental health, suicidality, depression and anxiety. YAM aims to raise awareness of mental health, risk and protective factors, and improve skills to deal with stress, including an emphasis on help-seeking. Following the YAM sessions, students participated in a one-hour KRAS (“scratch” in Dutch; Baetens et al., 2020) session. KRAS was developed to prevent NSSI, reduce stigma, and includes psychoeducation about NSSI (prevalence, functions, risk factors) alongside in-class interactive discussions (Baetens et al., 2020). The KRAS session also included information and discussion on the role of social media, (de-)stigmatization and help-seeking for NSSI. The content and procedures of YAM and KRAS were presented in manuals, which were followed by the two instructors who delivered the intervention to ensure fidelity to the methods. Structured adherence ratings were not registered. The instructors for the YAM and KRAS sessions in this study had professional backgrounds, either as licensed psychologists or as registered psychiatric nurses with clinical experience working with children and adolescents. The instructors received training and supervision from certified YAM educators.

In addition, psychoeducation and training were given to parents, that is, adolescents’ legal guardians, and school staff who were invited to participate in a brief webinar on NSSI. Expert health staff were further invited to a two-day educational workshop on NSSI and suicidality (Strong Schools against Suicidality and Self-injury; Brown et al., 2018; Groschwitz et al., 2017). For further information on these interventions, procedures and participants, see supplementary materials, S1.

### Measures

The survey included background questions about gender, age, country of birth, and parental employment and housing.

#### NSSI

To capture a range of aspects regarding NSSI, the survey included lifetime prevalence of NSSI from the Self-injurious Thoughts and Behavior Interview (SITBI-SF-SR, short form, self-report; Nock et al., 2007), which has been tested in Swedish adolescents (Zetterqvist et al., 2013), and NSSI behaviors and frequency from the Inventory of Statements about Self-injury (ISAS; Klonsky & Glenn, 2009), previously used in Swedish samples (e.g., Lindholm et al., 2011). (Lifetime NSSI prevalence is operationalized in the SITBI-SF-SR as a dichotomous item: “Have you ever actually engaged in NSSI?”. The ISAS behavioral checklist consists of eleven NSSI behaviors (the items regarding pinching and swallowing dangerous substances were omitted as they fall outside the definition of NSSI), and respondents indicate the number of times they have carried out each behavior (Klonsky & Glenn, 2009). In addition, items from the ISAS regarding past-year frequency, three-month frequency, and age of onset were included.

#### Difficulties in Emotion Regulation

Difficulties in Emotion Regulation were measured using the Difficulties in Emotion Regulation Scale (DERS-16; Bjureberg et al., 2016). In the present study, only the overall ER score was used, which is the sum score of 16 items rated on a five-point Likert scale thus yielding a score between 16 and 80, where higher score indicates greater ER difficulties. The original DERS-16 validation study was carried out using the Swedish translation, and the measure has been validated in Swedish adolescent samples (Monell et al., 2022). Cronbach’s alpha in the current study sample was 0.95, indicating excellent internal consistency reliability, while McDonald’s OmegaT was 0.96, also indicating excellent reliability.

#### Self-criticism

Measures of self-criticism were collected with the Self-Rating Scale (SRS; Hooley et al., 2010). The SRS comprises eight items rated on a 0 to 7 scale, with a higher total score reflecting a greater degree of self-criticism. The SRS total score yielded a Cronbach’s alpha of 0.88, and McDonald’s OmegaT of 0.92 in the current sample, indicating good reliability.

#### Health-Related Quality of Life

Health-Related Quality of Life (HRQoL) was measured using the KIDSCREEN-52, yielding an overall score and subscale scores for Physical Well-Being, Psychological Well-Being, Moods and Emotions, Self-Perception, Autonomy, Parent Relations and Home Life, Financial Resources, Peers and Social Support, School Environment, and Social Acceptability (Ravens-Sieberer et al., 2008). Swedish was one of the languages used in the original KIDSCREEN-52 validation study (Ravens-Sieberer et al., 2008). The overall score was not used in the present study, since HRQoL as a broader construct was not a target of the interventions. The Social Acceptability subscale had Cronbach’s alpha 0.72 and McDonalds OmegaT 0.76 in the present sample, indicating acceptable reliability. The other subscales had Cronbach’s alphas ranging between 0.83 and 0.93, and McDonald’s OmegaTs ranging between 0.87 and 0.96, indicating good to excellent reliability.

#### Mental Health-related Stigma

Mental health-related stigma was assessed using the Peer Mental Health Stigmatization Scale-Revised (PMHSS-R; McKeague et al., 2015). In the PMHSS-R, participants indicate their agreement with eleven statements regarding youth and mental health on a Likert-type scale from 1 (“disagree completely”) to 5 (“agree completely”). Two subscale scores are yielded, one for stigma awareness and one for stigma agreement. Stigma awareness refers to the perception of stigma in general, and stigma agreement to the degree of negative attitudes that the participants themselves acknowledge. Cronbach’s alphas for stigma awareness and stigma agreement in the study sample were 0.83 and 0.80, indicating good internal consistency reliability. McDonald’s OmegaTs were 0.88 and 0.85, respectively, also indicating good reliability.

#### Perceived Social Support

Perceived social support was measured using the Multidimensional Scale of Perceived Social Support (Zimet et al., 1988). The MSPSS measures perceived social support in the domains friends, family and significant other using a total of 12 items rated on a seven-point Likert scale. The scale has been validated in a Swedish sample (Ekback et al., 2013). The subscales friends, family and significant other had Cronbach’s alphas between 0.89 and 0.93, indicating good to excellent internal consistency reliability. McDonalds OmegaTs for friends, family and significant other were 0.94, 0.23, and 0.92, respectively.

#### Help-seeking

Finally, to gather adolescents’ perceptions of help-seeking, the three scales developed by (Schmeelk-Cone et al., 2012; Help-Seeking Acceptability at School, HSA; Adult Help for Suicidal Youth, AHSY; Reject Codes of Silence, RCS) were utilized. The three subscale scores are calculated from ten items, which participants answer on a scale ranging from 1 (“strongly disagree”) to 4 (“strongly agree”). HSA captures participants’ expectations toward seeking and receiving help, AHSY is intended to measure the perceived availability of adult support, and RCS measures the barriers participants perceive associated with seeking help in case of suicidality. On all three scales, higher scores reflect more positive attitudes towards support, help-seeking and disclosure. Cronbach’s alphas for HSA, AHSY and RCS in the study sample were 0.80, 0.76, and 0.62, while McDonald’s OmegaTs were 0.88, 0.77, and 0.72, respectively. Cronbach’s alpha thus indicates acceptable internal consistency reliability only for HSA and AHSY, while McDonald’s OmegaT indicates acceptable reliability for all three scales.

### Statistical Analysis

Frequencies, percentages, means, medians and standard deviations were calculated. All outcomes were evaluated using mixed effects models. Linear mixed effects models (LMMs) were used for continuous outcomes, generalized linear mixed effects models (GLMMs) for binary outcomes, and cumulative link mixed effects models (CLMMs) for ordinal outcomes. Data from three time points (baseline, post-intervention, and follow-up) were used, and only participants with complete data were included. Models were specified with measurement time point and group (intervention or control) as fixed effects (separately and in interaction) and with individual random intercepts, thus accounting for variance related to the experimental procedure as well as variance related to individual differences:

$${\rm{Value}} \sim {\rm{Time}}* {\rm{Group}}+(1|{\rm{participant}})$$

Fixed effects were evaluated using Analysis of Variance (ANOVA) with Type III Sums of Squares and *p*-values based on Satterthwaite degrees of freedom, or, where appropriate, the Wald test (type III). Significant interactions between the group and measurement time point were interpreted as potential effects related to the experimental procedure. Such interaction effects were further explored by pairwise comparisons of estimated marginal means (Tukey adjusted *p*-values). Partial Eta^2^ effect sizes and r^2^ explained variance (Nakagawa et al., 2012) were computed.

### Primary Outcomes

The effects of the intervention on NSSI onset were evaluated using a generalized linear mixed effects model (GLMM) specifying NSSI history (ever having engaged in) as dependent. Participants indicating previous NSSI at baseline were excluded in this analysis as their NSSI onset had already occurred. Survey items about having engaged in NSSI post-intervention and follow-up measurements were included as dependent variables.

NSSI three-month frequency was re-coded from text answers in SITBI to integers and then arranged in ordinal categories (0, 1, 2–3, 4–7, 8–13, 14–20, 21–30, or above 30 episodes). The NSSI frequency category at baseline, post-intervention, and follow-up measurements were then used as dependent for a cumulative link mixed effects model (CLMM; Christensen, 2023). Data from all participants in the study sample were included in this analysis.

### Secondary Outcomes

The effects of the intervention on social support, self-criticism, quality of life factors, stigma, help-seeking, and difficulties in emotion regulation were evaluated using linear mixed effects models (LMMs), specified similarly to the GLMM and CLMM models. One model per outcome variable was fitted, using maximum likelihood (ML) estimation.

Additionally, exploratory analyses of gender and NSSI history were conducted to investigate whether they moderated the primary effects of the intervention. Significant interaction effects suggest that the impact of the intervention varies depending on the levels of the included variable, indicating a moderation effect. For analyses including gender, only groups of participants identifying as male or female were included, as the group identifying as non-binary was too small.

Analyses were performed using R (version 4.4.0; R Core Team, 2020) and the *lme4* and *lmerTest* libraries (Bates et al., 2015; Kuznetsova et al., 2017). For ordinal outcomes, the *ordinal* R package (Christensen, 2023) was used.

## Results

Of the participants, 183 took part in data collection at all three time points and were included in the analysis. Table 2 summarizes descriptive data for all outcomes among these participants.

**Table 2:** Means and Standard Deviations for all Outcomes at Baseline, Post-Intervention and Follow-up for Intervention (*n* = 97) and Control Group (*n* = 86)

Outcome	Baseline		Post-intervention		Follow-up	
	Intervention	Control	Intervention	Control	Intervention	Control
NSSI Lifetime prevalence (single-item) (n %)	15 (15.5%)	22 (25.6%)	12 (12.4%)	24 (27.9%)	11 (11.3%)	20 (23.3%)
NSSI Lifetime prevalence (checklist)^a^ n (%)	39 (40.2%)	39 (45.3%)	-	-	-	-
NSSI Three-month prevalence	14 (14.43%)	21 (24.42%)	5 (5.15%)	16 (18.6%)	6 (6.19%)	8 (9.3%)
NSSI Onset (n)	-	-	2	6	3	0
Social support	*M* (*SD*)		*M* (*SD*)		*M* (*SD*)	
Friends	22.81 (5.6)	21.65 (5.31)	22.21 (5.53)	22 (6.05)	21.39 (5.97)	21.51 (5.86)
Family	24.34 (4.64)	22.77 (6.16)	23.42 (5.45)	22.71 (6.21)	22.81 (5.64)	22.59 (6.32)
Significant Other	23.84 (4.61)	23.57 (5.4)	22.88 (5.6)	23.43 (5.06)	22.95 (5.51)	22.84 (5.59)
Self-criticism	24.36 (11.82)	24.56 (11.94)	23.44 (12.43)	22.36 (11.72)	23.13 (11.79)	23.43 (11.96)
Health-Related Quality of life						
Physical Well-Being	17.31 (4.12)	16.9 (4.79)	18.07 (4.1)	17.55 (3.96)	18.13 (4.1)	17.19 (4.67)
Psychological Well-Being	23.35 (4.96)	22.65 (5.17)	23.26 (5.05)	22.94 (5.1)	23.36 (4.68)	22.95 (4.89)
Moods and Emotions	27.38 (6.5)	26.36 (7.23)	27.89 (6.56)	26.3 (6.82)	27.67 (6.52)	27.26 (6.62)
Self-Perception	17.24 (5.76)	16.44 (5.62)	17.65 (5.69)	17 (5.75)	17.82 (5.28)	16.65 (4.82)
Autonomy	20.18 (4)	19.23 (3.93)	20.07 (3.79)	19.66 (3.77)	20.03 (3.9)	19.7 (4.02)
Parent Relations and Home Life	26.32 (4.15)	25.69 (4.77)	25.92 (4.78)	25.41 (4.74)	25.57 (4.88)	24.97 (5.42)
Financial Resources	13.22 (2.24)	12.85 (2.75)	13.13 (2.43)	12.52 (2.96)	12.57 (3.08)	12.63 (3.17)
Peers and Social Support	23.12 (4.94)	23.2 (5.2)	23.37 (5.34)	23.34 (4.61)	23.23 (5.12)	22.8 (5.2)
School Environment	21.82 (4.93)	20.63 (5)	21.47 (5.05)	21.51 (4.82)	21.36 (5.29)	21.35 (4.73)
Social Acc. (Bullying)	13.45 (2.1)	13.48 (2.23)	13.49 (2.23)	13.47 (2.1)	13.57 (2.48)	13.38 (2.36)
Stigma						
Agreement	6.97 (2.67)	7.81 (3.23)	7.02 (3.38)	7.42 (3.41)	7.87 (4.2)	7.79 (3.5)
Awareness	14.84 (4.89)	15.88 (5.54)	14.42 (5.72)	14.8 (5.29)	15.54 (6.22)	14.05 (5.41)
Help-seeking						
Help-Seeking Acceptability	11.38 (2.86)	11.02 (2.68)	10.95 (3.16)	10.77 (2.97)	10.65 (3.38)	10.35 (3.5)
Adult Help for Suicidal Youth	9.29 (1.85)	8.9 (2.18)	9.05 (2.01)	8.72 (2.24)	8.88 (2.49)	8.97 (2.31)
Reject Codes of Silence	9.37 (1.91)	8.79 (2.1)	9.1 (1.76)	8.81 (1.87)	8.93 (1.94)	8.69 (1.83)
Difficulties in emotion regulation	33.57 (14.26)	38.33 (16.81)	31.13 (12.62)	36.86 (15.83)	31.58 (12.6)	34.48 (16.01)

^a^NSSI checklist (from the ISAS) data available only at baseline

### Primary Outcomes

Regarding the effect of the intervention on the onset of NSSI, a total of 11 participants with complete data (out of *n* = 183; five in the intervention group and six in the control group) reported having ever engaged in NSSI post-intervention and/or follow-up measurements, despite having answered negatively at baseline. The generalized linear mixed effects model did not yield a significant interaction between time and experimental condition (intervention or control; Wald test) *W*(2) = 0.235, *p* = 0.889.

**Fig. 2:**
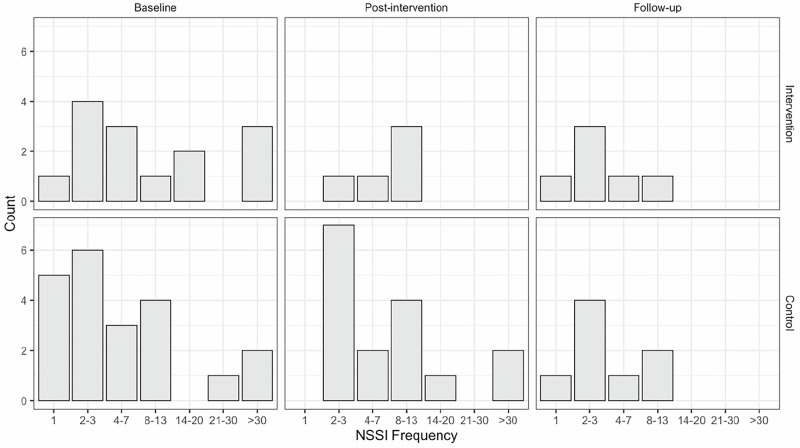
NSSI Three-month Frequencies at Baseline, Post-Intervention and Follow-up for Intervention and Control Group. *Note:* The frequencies show how often participants reported engaging in NSSI over the last three months. Responses indicating zero occurrences are not included in the figure. Frequencies are categorized as utilized in the analyses

**Fig. 3:**
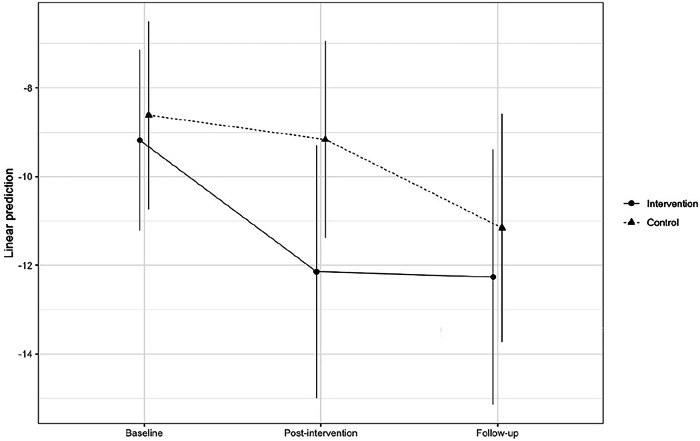
NSSI Three-month Frequency – Estimated Marginal Means for Control and Intervention Group. *Note:* Vertical lines indicate 95% confidence intervals of the estimates

In the case of the effect of the intervention on NSSI frequency, sixty-one participants (33.3%) had responded with values above zero on the item regarding having engaged in NSSI during the last three months at one or more of the three measurement time points (see Fig. 2). The cumulative link mixed-effects model showed a significant interaction effect between time and condition *W*(2) = 6.2561, *p* = 0.0438. Post-hoc pairwise comparisons (see Fig. 3 for estimated marginal means) showed that at the post-intervention measurement, the intervention group had an estimated score significantly different – lower than at the baseline measurement (*z* = 3.455, *p* = 0.0073) as well as lower than the control group at baseline (*z* = 3.010, *p* = 0.0313). This was also true for the follow-up measurement, where the intervention group differed from its baseline measurement (*z* = 3.628, *p* = 0.0039) and the control group at baseline (*z* = 3.125, *p* = 0.0220). However, at follow-up, a significant change had occurred in the control group, and the estimated score was significantly lower than the control group’s score at baseline (*z* = 3.914, *p* = 0.0013) as well as post-intervention (*z* = 3.068, *p* = 0.0263). This model yielded a Marginal *r*^2^ of 0.041 (Nakagawa; Conditional *r*^2^ was 0.940).

Running the NSSI frequency model and subsequent analysis of variance and including only the baseline and post-intervention measurements showed an even more articulated effect *W*(1) = 8.3749, *p* = 0.0038. In this case, post hoc pairwise comparisons showed that at the post-intervention measurement time point, the intervention group had an estimated score significantly lower than its baseline score (*z* = 3.780, *p* = 0.0009) as well as the control group at baseline (*z* = 3.205, *p* = 0.0074) and post-intervention (*z* = 2.772, *p* = 0.0285) measurement time points. This model had Marginal *r*^2^ 0.041 (Conditional *r*^2^ 0.959).

### Secondary Outcomes

Secondary outcomes were also analyzed, including health-related quality of life factors, stigma agreement and awareness, perceived social support, help-seeking, self-criticism and difficulties in emotion regulation. For the continuous outcomes modeled with linear mixed-effects models, significant interaction effects between condition and time of measurement were found in the case of Stigma Awareness (see Table 2). The effect size (Partial η^2^) for the interaction was 0.02, indicating a small effect. Pairwise comparisons (within Condition to keep the number of comparisons down) showed that the estimated mean scores of the control group were lower at follow-up compared to baseline (t(370) = 2.653, *p* = 0.226). Visual inspection suggested that the intervention group had a higher estimated mean score at follow-up compared to baseline, however, this difference was not statistically significant (*t*(370) = 1.075, *p* = 0.530). No significant interaction effects were found for the other secondary outcomes (see Table 3).

**Table 3:** Interaction Between Fixed Effects (Condition by Time of Measurement) for Secondary Outcomes

Outcome	F	DF1	DF2	*p*	Partial η^2^
Social support					
Friends	1.487	2	366	0.227	0.008
Family	2.231	2	366	0.109	0.012
Significant Other	0.686	2	366	0.504	0.004
Self-Criticism	0.517	2	366	0.597	0.003
Health-Related Quality of Life					
Physical Well-Being	0.640	2	366	0.528	0.003
Psychological Well-Being	0.205	2	366	0.814	0.001
Moods and Emotions	0.984	2	366	0.375	0.005
Self-Perception	0.515	2	366	0.598	0.003
Autonomy	0.578	2	366	0.561	0.003
Parent Relation and Home Life	0.021	2	366	0.98	0
Financial Resources	1.319	2	366	0.269	0.007
Peers and Social Support	0.222	2	366	0.801	0.001
School Environment	2.487	2	366	0.085	0.013
Social Acc. (Bullying)	0.177	2	366	0.838	0.001
Stigma					
Agreement	1.413	2	366	0.245	0.008
Awareness	3.869	2	366	0.022	0.021
Help-Seeking					
Help-Seeking Acceptability	0.084	2	366	0.92	0
Adult Help for Suicidal Youth	1.259	2	366	0.285	0.007
Reject Codes of Silence	0.806	2	366	0.447	0.004
Difficulties in Emotion Regulation	1.461	2	366	0.23	0.008

### Exploratory Analyses

Subsequent models, including gender and baseline NSSI history as covariates, yielded interaction effects that were not found in the overall models. However, models that included gender showed significant interaction effects (Time x Condition x Gender) for outcomes Bullying (KIDSCREEN) *F*(2356) = 5.530, *p* = 0.004, partial η^2^ = 0.030, Stigma Awareness *F*(2356) = 6.270, *p* = 0.002, η^2^ = 0.034 and Help-seeking: Reject codes of silence (RCS) *F*(2356) = 6.045, *p* = 0.003, partial η^2^ = 0.033. For these outcomes, models were run separately for each gender, specifying an interaction between time and condition. Pairwise comparisons were carried out within Condition for models with significant interaction effects.

For Bullying, the analyses showed a significant interaction effect among girls *F*(2212) = 3.693, *p* = 0.027, partial η^2^ = 0.034, but not among boys *F*(2144) = 2.104, *p* = 0.126. Pairwise comparison of estimated means among girls showed a significant increase between baseline and follow-up in the intervention group *t*(216.08) = −2.415, *p* = 0.044. For Stigma Awareness, there was a significant interaction effect among boys *F*(2144) = 6.640, *p* = 0.002, partial η^2^ = 0.084 that was not seen among girls *F*(2212) = 2.124, *p* = 0.122. Pairwise comparisons of estimated means among boys (within Condition) showed a significant decrease in the control group from baseline to post-intervention *t*(148.11) = 2.607, *p* = 0.027 and from baseline to follow-up *t*(148.11) = 2.679, *p* = 0.022. For RCS, there was a significant interaction effect among boys *F*(2144) = 5.1236, *p* = 0.007, partial η^2^ = 0.066 that was not seen among girls *F*(2212) = 0.968, *p* = 0.382. Pairwise comparisons showed a significant decrease in the intervention group between baseline and follow-up *t*(148.11) = 3.711, *p* = 0.0008 and between post-intervention and follow-up *t*(148.11) = 2.598, *p* = 0.0277.

Models including baseline NSSI history as covariate showed significant interaction effects (Time x Condition x NSSI history) for outcomes Social Support: Friends *F*(2, 366) = 3.379, *p* = 0.035, partial η^2^ = 0.018, KIDSCREEN Peers and Social Support *F*(2, 366) = 3.929, *p* = 0.020, partial η^2^ = 0.021, and KIDSCREEN School Environment *F*(2, 366) = 3.099, *p* = 0.046, partial η^2^ = 0.017. Separate models were run including participants with or without a history of NSSI at baseline.

For Social Support: Friends, analyses revealed a significant interaction effect among those without a history of NSSI *F*(2292) = 3.618, *p* = 0.0280, partial η^2^ = 0.024 that was not seen among participants with a history of NSSI *F*(2,74) = 1.058, *p* = 0.352. Pairwise comparisons showed a significant decrease in the intervention group between baseline and follow-up *t*(296,06) = 3.431, *p* = 0.002, partial η^2^ =. For KIDSCREEN Peers and Social Support, there was an interaction approaching significance in the NSSI group *t*(2,74) = 2.813, *p* = 0.066, partial η^2^ = 0.071. Post-hoc pairwise comparisons showed a significant increase between baseline and post-intervention for the intervention group *t*(78.23) = 2.461, *p* = 0.042. For KIDSCREEN School Environment, there was a significant interaction effect in the group with NSSI history *F*(2,74) = 5.003, *p* = 0.009, partial η^2^ = 0.119 that was not seen in the other group *F*(2292) = 1.884, *p* = 0.154. Subsequent pairwise comparisons showed significant increases between baseline and follow-up *t*(78.23) = 3.314, *p* = 0.004 and between post-intervention and follow-up *t*(78.23) = 2.531, *p* = 0.035 in the control group.

## Discussion

Mental health problems and NSSI in adolescents are serious concerns, and more information is needed on how to address and prevent these issues. In this study, the effects of interventions targeting mental health and NSSI, delivered to students, parents, teachers, and healthcare staff, were examined using a cluster-randomized experimental design. The study found that the whole-school preventive intervention was associated with a reduction in NSSI frequency. The estimated frequency was significantly decreased in the intervention group but not in the control group when comparing baseline and post-intervention measurements. No evidence was found to support a change in NSSI onset. The whole-school intervention also had an effect on self-reported stigma awareness. No significant effects were found on attitudes toward help-seeking, perceived social support, health-related quality of life, self-criticism, or emotion regulation. Explorative analyses further showed gender-specific effects on bullying, stigma awareness, and help-seeking, along with effects specific to adolescents with or without a history of NSSI regarding friendship and social support from friends. Whole-school interventions addressing mental health and NSSI in adolescents can be effective for influencing NSSI and stigma, with potential additional specific effects for subgroups of adolescents,

### NSSI Onset and Frequency

This study contributes to the still limited body of research investigating the effects of school-based universal prevention targeting NSSI, and aligns with earlier findings showing that addressing NSSI in educational settings does not seem to have iatrogenic effects (Baetens et al., 2020; Baetens et al., 2024; Muehlenkamp et al., 2010). Like Baetens et al. (2024) and Zare et al. (2023), it was found that the current school-based prevention reduced NSSI frequency. Reduction of NSSI frequency in adolescents with NSSI is important as repetitive NSSI has negative long-term outcomes (Daukantaite et al., 2020b).

There is an ongoing debate about whether the frequency of NSSI is an appropriate outcome for treatment and prevention studies. The results suggest that versatility (defined as the number of different methods of self-injury) alone (Turner et al., 2013), or in combination with frequency (Ammerman et al., 2020), might be a better indicator of NSSI severity and hence preferable. Despite this, frequency remains the most commonly used behavioral outcome related to NSSI (Calvo et al., 2022; Witt et al., 2021) and is associated with the risk of suicide attempts (Brausch & Boone, 2015). Concerning changes in NSSI frequency, Muehlenkamp et al. (2023) identified this to be significantly associated with a change in suicidal ideation; however, their study was observational, and the mechanisms of these changes were not investigated. Based on earlier research, lower NSSI frequency thus has the potential to reduce the risk of suicidal behavior (Prinstein et al., 2008) and can be an important step toward NSSI cessation (Kiekens et al., 2017).

The estimated NSSI frequency in the intervention group significantly decreased from baseline to post-intervention and was at post-intervention significantly lower than that in the control group. However, this difference did not persist at the follow-up measurement, as, at that time point, the frequency in the control group was also lower and significantly different from its baseline estimate. This might be explained by the follow-up measurement being carried out just after students returned from their two-month summer holiday, something which reasonably affected participants in both the intervention and control group. There is substantial evidence indicating increased self-reported mental health (Kristjansdottir et al., 2013), lower rates of mental health-related hospital admissions (Radhakrishnan et al., 2023; Slaunwhite et al., 2019), and reduced rates of self-harm (Jack et al., 2023) during school holiday periods. Furthermore, in qualitative interviews, students from the present study sample identified academic stress as a major contributor to mental ill-health (Aspeqvist, Münger, et al., 2024), which would have been absent during the holiday.

We did not, however, detect a change in NSSI onset as an effect of the intervention. The sample size can reasonably explain this, as the participation rate was low, and it is a known fact that many adolescents who engage in NSSI begin at an earlier age (Aspeqvist, Andersson, et al., 2024; Plener et al., 2015). Thus, to detect delay or actual prevention of NSSI, one would need to deploy earlier intervention and study considerably larger sample sizes. For example, Baetens and colleagues (2024) focused on a community of 11–14-year-olds and reported a significant reduction in NSSI frequency after a short universal preventive intervention.

### Stigma, Help-seeking and Social Support

Further, results indicated an effect on Stigma Awareness. There was no significant change in the intervention group, but a significant decrease in the control group and exploratory analyses showed that this effect was driven by a decrease among boys in the control group. The effect size was small; however, small effect sizes can have large impacts when interventions are delivered to large populations (Funder & Ozer, 2019; Rosenthal, 1990; Werner-Seidler et al., 2021). The Stigma Awareness subscale of the PMHSS-R is built from items with statements such as “Most people look down on children who visit a counselor because they have emotional or behavioral problems.” where higher scores indicate agreement with the statement. The designers of the PMHSS-R describe the Awareness subscale as intended to measure awareness of prevailing social stigma and differentiate this from personally held stigmatizing beliefs (McKeague et al., 2015). Stigma surrounding mental health and NSSI is something that is addressed in both the KRAS and YAM interventions that were delivered to adolescents in the current study. As mental health stigmatization is a real and widespread phenomenon of great concern (Thornicroft et al., 2022), a reasonable conclusion is that the intervention group ended up with increased awareness of the stigma associated with mental health problems generally and NSSI specifically, which expressed itself as a more realistic, albeit somewhat darker, view of social stigma in comparison to the control group at follow-up. An earlier qualitative study on participants’ experiences with the intervention (Aspeqvist, Münger, et al., 2024) revealed that adolescents reported a greater understanding and compassion for their peers following the intervention. Since stigma poses a barrier to help-seeking (Barrow & Thomas, 2022) and the disclosure of NSSI (Rosenrot & Lewis, 2018), fostering more understanding environments may benefit those needing social support and mental health services.

The current study found no significant differences between intervention and control conditions on help-seeking, social support, self-criticism, and emotion regulation, even though the importance of help-seeking, for example, is emphasized throughout YAM. These null results do not confirm earlier research on school-based interventions (Baetens et al., 2024; Cipriano et al., 2021), which found effects on attitudes toward help-seeking and emotion regulation, for example. YAM has previously shown effects on help-seeking (Lindow et al., 2020). Due to the low participation rate in the current study, it might be underpowered to document effects. In qualitative evaluations of the intervention (Aspeqvist, Münger, et al., 2024), participants reported being aware of the importance of help-seeking message, which was, however, not reflected in the quantitative measures included here. Interestingly, a “help-seeking vs. autonomy dilemma” was found, where participants reported being aware of the need to seek help from adults in matters related to mental health, and, at the same time, expressing a need to balance this against their wish for autonomy and coping for themselves. This implies that help-seeking may be a more complex construct for this age group, which needs to be considered when interpreting the results. As Foulkes and Stapley (2022) pointed out, listening to adolescents’ perspectives and suggestions for improvements and combining qualitative and quantitative measures in evaluating preventive interventions has the potential for a more nuanced understanding of results.

### Gender and NSSI History as Moderators

Explorative analyses yielded significant interaction effects when gender and baseline NSSI history were included in the linear mixed effects models. Thus, the whole-school preventive intervention appeared to have effects that were differentiated by these factors. Regarding stigma awareness, it became clear that the overall effects were specific to and more pronounced in male participants. This has potential importance, as an environment with less stigmatization might increase help-seeking among boys, a group that is otherwise less likely to seek help (Barrow & Thomas, 2022; Haavik et al., 2019).

In the intervention group, there was also a positive effect on bullying specific to girls, indicating that they experienced less fear of others, being made fun of, or being bullied. A lower level of bullying and negative social interaction can have a large impact, as bullying is linked to a range of adverse mental health outcomes longitudinally (Lereya et al., 2015) and also, as negative social interaction has been linked to increased NSSI urges (Haliczer & Dixon-Gordon, 2023).

Further, there was a reduction in norms on not talking to others about suicidality among boys. There are gender differences in suicide among adolescents and young adults. Suicide attempts are more common in females, but suicide rates are higher in males. (Miranda-Mendizabal et al., 2019). Breaking the silence among boys is one step forward in suicide prevention. As described above, the YAM program aimed at reducing suicidality and was effective in a large randomized controlled trial (Wasserman et al., 2015).

Effects found to be differentiated by NSSI in the present study are difficult to interpret. Participants in the intervention group without a history of NSSI seem to have experienced a decrease in social support from friends as measured with MSPSS. Estimates suggested a potential increase in social support among participants with NSSI in the intervention group, but this difference was not statistically significant. On the KIDSCREEN Peers and Social Support subscale, reports from participants with a history of NSSI point to an increase in their friendships as an effect of the intervention. Social support is a protective factor for NSSI (McEvoy et al., 2023) and is important in NSSI cessation (Andersson et al., 2024; Simundic et al., 2024). Further, estimates point to an improvement in the school situation (as measured by the KIDSCREEN School Environment subscale) for those with NSSI in the control group. A possible explanation for this is that some change took place locally at one of the schools between measurements. On the one side, the overall aim of universal interventions is to achieve population-level effects. However, the fact that group-specific effects emerged can also be interpreted as support for the suggestion that preventive interventions may not be best investigated solely in terms of effects on whole sample means but by potentially looking further into effects on specific subgroups (Birrell et al., 2025; Greenberg & Abenavoli, 2016; Supplee et al., 2013). However, caution needs to be taken in interpreting these results due to the explorative nature of the analysis. Future research could further investigate the nature of subgroup-specific effects on universal prevention targeting NSSI.

The current study adds to the small body of research documenting effects of preventive interventions targeting NSSI. The results show that whole-school intervention of the type investigated here can have an effect on NSSI frequency in an adolescent population. Reduction in NSSI frequency was recently reported in another intervention combined with KRAS (Baetens et al., 2024). While YAM has documented effects related to suicide attempts and ideation (Wasserman et al., 2015), it has not previously been part of an intervention targeting NSSI. This study confirms earlier research indicating that it is possible to influence adolescents’ perceptions of stigma (Lanfredi et al., 2019). Finally, this study acknowledges the potential importance of examining effects in specific subgroups (Laurenzi et al., 2023).

### Strengths and Limitations

A key strength of this study is its randomized controlled design, which enables conclusions about the specific effects of the intervention. Although a larger sample size would have been ideal, the current sample is sufficient to statistically verify a range of experimental effects. The study was carried out in a real-world setting—ordinary Swedish secondary schools—and this enhances the generalizability of the results to the broader population. Another advantage is the extensive survey battery, which allowed for analysis of effects across various relevant constructs.

However, there are some limitations to the study that need to be mentioned. One limitation is the participation rate, which was 25.3% of eligible adolescents. This was partly affected by the ethical requirement for both caregivers to provide active, informed consent. Moreover, the study was initiated during the last phase of the COVID-19 pandemic, which also affected recruitment; there were no school closures in Swedish compulsory (primary and lower secondary) schools (Lindblad et al., 2021), but social distancing affected procedures during the recruitment phase. While we are not aware of systematic effects on participation, the study sample may differ in some respects from the total eligible population. There could be a potential bias in adolescents who participated in the study, as caregivers with an interest in the topic and research in general may have been more likely to give consent. Regrettably, since publicly available statistics on the participating schools are limited and differ from the sample data regarding question types, this prevents an analysis of selection bias. However, eligible participants and their caregivers were unaware whether their school was assigned to the intervention or control group (the control group received the same interventions at a later stage). Thus, there is no reason to suspect different participation patterns between the groups. The achieved attrition rates of 24.4% at post-measurement and 13.5% at follow were relatively low and, while not optimal, limit the potential effects of attrition bias. Demographic data in Table 1 show that regarding sex, living with both parents, and country of origin, attrition seems not to have caused differences between the full (baseline) sample and the study sample (participants with data from all three measurements). Further, while Cronbach’s alpha indicated acceptable internal consistency reliability, the low OmegaT for the social support from family subscale indicates that the subscale might be reflecting more than one single construct in the study sample. Previous research points to challenges with retrospectively self-reporting behavior like NSSI (Daukantaite et al., 2020a), seemingly affecting the present sample as reported lifetime NSSI prevalence decreases across measurements. There was no measurement of cognitions and attitudes about NSSI before and after the intervention, which is a limitation. This would have allowed for more participant responses on NSSI-related variables, including those with no NSSI experience. Another limitation is that student attendance for each session was not recorded, nor were adherence ratings for fidelity to the methods used. As the study was cluster randomized with few clusters (i.e., schools), changes at the school level might have influenced the findings. No such changes were, however, brought to the attention of study staff. Moreover, as described above, seasonal effects have been documented on various mental health constructs, and as the follow-up measurement was carried out shortly after the summer holiday, such effects might have influenced the data. Lastly, as the study had a single treatment arm with all components of the whole-school intervention, study data do not allow for differentiating the effects of the components.

### Recommendations

Proactively addressing mental health in educational practice can help promote healthy adolescent development, and further research is needed on whole-school prevention strategies targeting NSSI and mental health. Ideally, samples should be considerably larger, as this would allow for detecting smaller effects and further investigation into what predicts beneficial responses for whom. Whole-school prevention studies should preferably be conducted with modular approaches, as researchers could determine which components contribute to the intended outcomes. Further research is also encouraged on gender-specific effects and the underlying mechanisms behind potential differences in outcomes, as needs may be met to varying degrees across genders by universal preventive interventions.

## Conclusion

Mental health issues and NSSI in adolescents are areas of concern. Support for mental health prevention is growing; however, the effectiveness of school-based prevention needs to be studied further. This study aimed to investigate the effects of a whole-school preventive intervention on NSSI onset and frequency as well as a range of secondary mental health-related outcomes. The study findings showed that the whole-school preventive intervention reduced the frequency of NSSI among adolescents. Furthermore, such an intervention can influence awareness of societal mental health-related stigma. Lastly, the findings indicate that the intervention had effects specific to subgroups, such as gender, within the studied population.

## Supplementary information

SUPPLEMENTARY MTRLS
