# Supplementary material for: Effects of a Whole-School Prevention Program Targeting Mental Health and Nonsuicidal Self-Injury in Swedish Adolescents: A Cluster-Randomized Experimental Study with Longitudinal Follow-Up
Source: J Youth Adolesc. 2025 Sep 11;55(4):795–813. doi: 10.1007/s10964-025-02251-3 (PMC13076508; doi:10.1007/s10964-025-02251-3)
Supplement: Supplementary file 1 — SUPPLEMENTARY MTRLS [file 10964_2025_2251_MOESM1_ESM.docx]

**Effects of a Whole-school Prevention Targeting Mental Health and Nonsuicidal Self-Injury in Swedish Adolescents: A Cluster-Randomized Experimental Study with Longitudinal Follow-Up**

Aspeqvist, Erik; Korhonen, Laura; Dahlström, Örjan; Andersson, Hedvig; Baetens, Imke; Plener, Paul; Zetterqvist, Maria.

**SUPPLEMENTARY MATERIALS**

**Supplement S1: Descriptions of whole-school interventions, procedures and participants**

## Adolescents

### Youth Aware of Mental-health Program (YAM)

The Youth Aware of Mental Health Programme (YAM) was developed for the Saving and Empowering Young Lives in Europe (SEYLE) study (Wasserman et al., 2012; 2015). It is a

manualised, universal intervention targeting students ages 13-17 years, mainly in the lower secondary school ages. YAM is delivered in the classroom with pedagogical material related to mental health posted on the classroom walls. Students are also given a booklet to take home. Five sessions are delivered over a three week period, which includes three hours of role-play sessions combined with two interactive lectures about mental health at the beginning and end of the intervention. YAM aims to raise mental health awareness, and knowledge about risk and protective factors associated with suicide, and depression and anxiety. It further aims to enhance skills needed to deal with adverse life events, stress, and suicidal behaviours. Further emphasis is on peer support and help-seeking when addressing mental health. The YAM programme was delivered in classrooms in participating schools by instructors trained in the methodology and manual.

### KRAS

KRAS is a one-session classroom module for students that covers topics relevant to the prevention of NSSI. It was developed, piloted and tested by Baetens (2020; 2024) and includes basic NSSI knowledge (prevalence, functions, risk factors), the role of social media, (de-)stigmatization of NSSI and help-seeking for NSSI. The module includes a documentary where young adults with lived experience of NSSI talk about what was helpful for them in the process toward recovery. A guided classroom discussion follows the documentary, where students and instructors discuss topics such as self-care, advice for adolescents engaging in NSSI (help-seeking is emphasized), how to handle NSSI in social media and possible contagion of NSSI, and also how to help a friend who self-injures.

#### Procedure and Participants

To be eligible for participation, students needed to be enrolled in a regular school class, specifically in grades seven or eight, following the standard Swedish curriculum. In the six participating schools, 1,054 students were eligible to participate in the study. Of these, 267 agreed to take part after receiving informed written consent from both caregivers, and 266 proceeded to baseline data collection. The calculated participation rate was 25.3%.

## Caregivers

### Brief Online Psychoeducation on Nonsuicidal Self-Injury for Caregivers

The online psychoeducation on nonsuicidal self-injury (NSSI) for caregivers was developed for the current project. It consisted of a 45-minute film with psychoeducation. The content included auditive information delivered by the last author (MZ) on the definition of NSSI, prevalence, and its functions, together with information on risk and protective factors, common comorbidities and the relationship between NSSI and suicidality. Further, it included information on how to increase stability through everyday routines. It also contained tips on how caregivers can talk to their child about NSSI, and some information on professional help.

#### Procedure and Participants

All caregivers of approximately 1,000 eligible adolescents in the participating lower secondary schools’ seventh and eighth grades were given written information about the study. The caregivers who gave written consent to participate received an e-mail containing a link to the online psychoeducation materials. In total, 569 caregivers gave active informed consent. Of these, 102 (17.9%) caregivers began taking part in the online psychoeducation on NSSI. Of these, 68 (66.7%) completed the psychoeducation and responded to the evaluative questions. Eighteen (26.5%) were men and 50 (73.5%) were women. Sixty-two (91.2%) were born in Sweden, 51 (75%) had a university/college education, seven (10.3%) had theoretical high school education, eight (11.8%) had attended a vocational high school program, and two (2.9%) had compulsory junior high.

## Teachers

### Brief Online Psychoeducation on Nonsuicidal Self-Injury for Teachers

The online psychoeducation on NSSI for teachers consisted of a 45-minute film developed for the current project with information about NSSI in adolescents presented orally by the last author (MZ). Questions and written summaries were also presented on the screen. Information on NSSI included the definition, prevalence, and functions of NSSI. Also, different risk and protective factors were presented, as well as common comorbidities and the relationship between NSSI and suicidality. Furthermore, information on how teachers can approach and talk to adolescents about NSSI, how teachers can help in the educational setting, NSSI cessation and recovery, and when and how caregivers and mental health services can be contacted were covered.

#### Procedure and Participants

Teachers were given written information and signed informed consent forms if they were interested in participating. Those who provided written consent were emailed a link to access the online psychoeducational materials. Of approximately 200 eligible teachers from the participating lower secondary schools, 159 gave active, informed consent to participate. Of these, 64 (40.3%) teachers started taking part in the online training, and 57 completed all data. Of these, 26 (45.6%) were men, and 31 (54.4%) were women. Fifty (87.7%) worked as teachers. Other participants were assistants or resource staff.

## School Health Staff

### Two-day Workshop on Nonsuicidal Self-Injury and Suicidality for School Staff

The content of the workshop was based on the gatekeeper program developed for the Strong Schools Against Suicidality and Self-injury project in Germany (Brown et al., 2018; Groschwitz et al., 2017). The material was translated and adapted to a Swedish context. The workshop was delivered during two consecutive days via PowerPoint, and films, and also included discussions, and some role play. Day one covered facts about suicidality and NSSI with information on definitions, prevalence, age of onset, and developmental course. Theories and models for NSSI and suicidality in adolescents were presented. Further, recommendations for how to react to and talk about NSSI and suicidality were presented and discussed, with an emphasis on validation. Basic information on emotions and emotion regulation was included, together with problem-solving and skills for coping with stress. A model for therapeutic assessment was presented, examining possible triggers, thoughts, emotions, and behaviors, as well as the short- and long-term consequences of NSSI. Day two consisted of risk assessment and legislation related to NSSI and suicidality. Suggestions for school protocols were discussed and recommendations for working together with caregivers were also presented.

***Procedure and Participants***

An offer to take part in the two-day workshop was given to principals and school health staff, such as nurses, psychologists, and counsellors and other staff, such as teachers’ coaches, assistants, and mentors in the participating schools. The workshop was delivered five times with five to 13 participants per workshop (*M* = 9.0, *SD* = 3.5) during January 2022 to October 2022. A total of 45 participants gave informed consent and took part in the workshop. We do not have data on all eligible school staff, since schools and staff could decide for themselves how many would take part in the workshop.

## Adolescents and Caregivers

### Emotion Regulation Skills Training for Adolescents and Caregivers

The emotion regulation skills training consisted of five sessions á two hours used has been piloted previously in clinical groups of adolescents and parents (Holmqvist Larsson et al., 2020). The skills training was voluntary and offered to adolescents and parents after school hours. Licensed psychologists with clinical experience of emotion regulation skills training and adolescent mental health conducted the training.

#### Procedure and Participants

A total of 267 adolescents (13-15 years in grades 7 and 8), and both their caregivers provided written informed consent in the larger preventive study, in which an opportunity to participate in a voluntary emotion regulation skills training for adolescents and parents after school hours was included. Of these 267 families, 11 reported interested in participating in the skills training. Four families dropped out before the skills training started. Two separate emotion regulation skills training groups were thus conducted for seven families with seven adolescents. One skills training was delivered at a school in May-June 2022 with five adolescents and six parents. The other group was held at a central location in November-December 2022 with two adolescents and two parents. One family dropped out after one session due to logistical difficulties.

## References

Baetens, I., Decruy, C., Vatandoost, S., Vanderhaegen, B., & Kiekens, G. (2020). School-Based Prevention Targeting Non-Suicidal Self-injury: A Pilot Study. *Frontiers in psychiatry*, *11*, 437. <https://doi-org.e.bibl.liu.se/10.3389/fpsyt.2020.00437>

Baetens, I., Van Hove, L., Azadfar, Z., Van Heel, M., & Soyez, V. (2024). The Effectivity of a School-Based Early Intervention Targeting Psychological Complaints and Non-Suicidal Self-Injury in Adolescents. *Journal of clinical medicine*, *13*(7), 1852. <https://doi-org.e.bibl.liu.se/10.3390/jcm13071852>

Brown, R. C., Straub, J., Bohnacker, I., & Plener, P. L. (2018). Increasing Knowledge, Skills, and Confidence Concerning Students' Suicidality Through a Gatekeeper Workshop for School Staff. *Frontiers in psychology*, *9*, 1233. <https://doi-org.e.bibl.liu.se/10.3389/fpsyg.2018.01233>

Groschwitz, R., Munz, L., Straub, J., Bohnacker, I., & Plener, P. L. (2017). Strong schools against suicidality and self-injury: Evaluation of a workshop for school staff. *School psychology quarterly : the official journal of the Division of School Psychology, American Psychological Association*, *32*(2), 188–198. <https://doi-org.e.bibl.liu.se/10.1037/spq0000185>

Holmqvist Larsson, K., Andersson, G., Stern, H., & Zetterqvist, M. (2020). Emotion regulation group skills training for adolescents and parents: A pilot study of an add-on treatment in a clinical setting. *Clinical child psychology and psychiatry*, *25*(1), 141–155. <https://doi-org.e.bibl.liu.se/10.1177/1359104519869782>

Wasserman, C., Hoven, C. W., Wasserman, D., Carli, V., Sarchiapone, M., Al-Halabi, S.,

Apter, A., Balazs, J., Bobes, J., Cosman, D., Farkas, L., Feldman, D., Fischer, G., Graber, N.,

Haring, C., Herta, D. C., Iosue, M., Kahn, J. P., Keeley, H.,…Postuvan, V. (2012). Suicide

prevention for youth--a mental health awareness program: lessons learned from the Saving

and Empowering Young Lives in Europe (SEYLE) intervention study. *BMC Public Health*,

*12*, 776. <https://doi.org/10.1186/1471-2458-12-776>

Wasserman, D., Hoven, C. W., Wasserman, C., Wall, M., Eisenberg, R., Hadlaczky, G.,

Kelleher, I., Sarchiapone, M., Apter, A., Balazs, J., Bobes, J., Brunner, R., Corcoran, P.,

Cosman, D., Guillemin, F., Haring, C., Iosue, M., Kaess, M., Kahn, J. P.,…Carli, V. (2015).

School-based suicide prevention programmes: the SEYLE cluster-randomised, controlled

trial. *Lancet*, *385*(9977), 1536-1544. <https://doi.org/10.1016/S0140-6736(14)61213-7>
